# Prognostic Significance and Immunological Role of FBXO5 in Human Cancers: A Systematic Pan-Cancer Analysis

**DOI:** 10.3389/fimmu.2022.901784

**Published:** 2022-06-03

**Authors:** Peng Liu, Xiaojuan Wang, Lili Pan, Bing Han, Zhiying He

**Affiliations:** ^1^ Institute for Regenerative Medicine, Shanghai East Hospital, School of Life Sciences and Technology, Tongji University, Shanghai, China; ^2^ Shanghai Engineering Research Center of Stem Cells Translational Medicine, Shanghai, China; ^3^ Shanghai Institute of Stem Cell Research and Clinical Translation, Shanghai, China; ^4^ Department of Pharmacy, Minhang Hospital, Fudan University, Shanghai, China

**Keywords:** FBXO5, bioinformatics, pan-cancer, prognosis, immunity, tumor microenvironment

## Abstract

F-box protein 5 (FBXO5), an essential subunit of the ubiquitin protein ligase complex, is increasingly recognized to exhibit important biological effects in regulating tumor occurrence and progression. The present research was intended to systematically investigate the latent roles of FBXO5 in prognosis and immunological function across cancers. Pan-cancer analyses of FBXO5 were performed based upon publicly available online databases, mainly including the Cancer Genome Atlas (TCGA), Genotype-Tissue Expression (GTEx), UCSC Xena, cBioPortal, and ImmuCellAI, revealing the possible relationships between FBXO5 and prognosis, DNA methylation, tumor microenvironment (TME), infiltration of immune cells, immune-related genes, immune checkpoints, tumor mutation burden (TMB), and microsatellite instability (MSI). The results suggested that FBXO5 was expressed at a high level in numerous tumor cell lines with significant upregulation in most cancers as opposed to normal tissues. Of note, elevated expression of FBXO5 was significantly related to an unfavorable prognosis in many cancer types. Furthermore, DNA methylation and TME were confirmed to display evident correlation with the expression of FBXO5 in several malignancies. Moreover, FBXO5 expression was remarkably positively correlated with the levels of infiltrating Treg cells and Tcm cells in most tumors, but negatively correlated with tumor-infiltrating CD8^+^ T cells, NK/NKT cells, and Th2 cells. Meanwhile, FBXO5 was demonstrated to be co-expressed with the genes encoding immune activating and suppressive factors, chemokines, chemokine receptors, and major histocompatibility complex (MHC). Immune checkpoints, TMB, and MSI were also overtly associated with FBXO5 dysregulation among diverse kinds of cancers. Additionally, the enrichment analyses showed close relationships between FBXO5 expression and the processes related to cell cycle and immune inflammatory response. These findings provided a detailed comprehension of the oncogenic function of FBXO5. Because of its crucial roles in cancer immunity and tumorigenesis, FBXO5 may serve as a novel prognostic indicator and immunotherapeutic target for various malignancies.

## Introduction

Malignant tumor poses a threat to global public health as a leading cause of human death and the main hazard factor reducing people’s quality of life, and so far, there is still a lack of absolutely effective treatment for cancer (1). Although early screening and surgery make heavy contributions to decreasing the incidence and mortality of malignancies, the prognosis and survival rate of most cancers remain unsatisfactory due to their characteristics of metastasis, recurrence, and heterogeneity (2). Tumor microenvironment (TME), containing various immune cells, stromal cells, and extracellular matrix, exhibits pivotal effects on tumor invasion and metastasis, cancer immunity, and clinical outcomes (3, 4). In recent years, immunotherapy has gradually become a prominent strategy for tumor treatment, especially immune checkpoint blockade therapy. Immune checkpoint inhibitors, such as CTLA-4-, PD-1-, and PD-L1-blocking antibodies, have been approved for the standard therapy in different malignancies (5). Nevertheless, the objective response rate remains minimal in many cancer patients receiving the same therapy (5, 6). Therefore, it is full of prospects to discover novel immunotherapeutic targets by analyzing gene expression in pan-cancer and exploring its correlations with clinical prognosis and tumor immunity.

F-box protein 5 (FBXO5), also referred to as early mitotic inhibitor-1 (EMI1), encodes a member of the F-box protein family and functions as an essential cell cycle regulating gene, which modulates the progression to S-phase and mitosis *via* the mechanism of blocking the anaphase-promoting complex (APC) (7, 8). According to previous reports, overexpression of FBXO5 produces chromosome instability and mitotic disorder, possibly resulting in the tumorigenesis in ovarian clear cell carcinoma (9), esophageal squamous cell carcinoma (10), breast carcinoma (11), and hepatocellular carcinoma (12). Existing evidence has suggested that FBXO5 affects tumor prognosis and clinical phenotypes. FBXO5 accumulation is tightly related to mitotic abnormalities including centrosome overduplication and aberrant spindle formation, which cause the emergence of tetraploidy in ovarian clear cell carcinoma (9). Moreover, elevated expression of FBXO5 is significantly correlated with an unfavorable prognosis among patients suffering from esophageal squamous cell carcinoma (10) and hepatocellular carcinoma (12). In addition, FBXO5 exhibits a pro-proliferative effect in breast cancer tissues through PI3K/Akt signaling pathway, while PI3K inhibitor can reduce FBXO5 expression and arrest cell growth (11). Based upon these findings, FBXO5 may perform an integral function in cell cycle abnormalities and the disruption of genomic stability, both of which can enhance tumor growth (13). At present, specific studies on FBXO5 in tumors appear to be restricted to certain human cancers, but lack of systematic pan-cancer investigation (9–12). In consequence, it is urgent to elucidate the significance and role of FBXO5 expression and alteration across different cancers.

**Figure 1:**
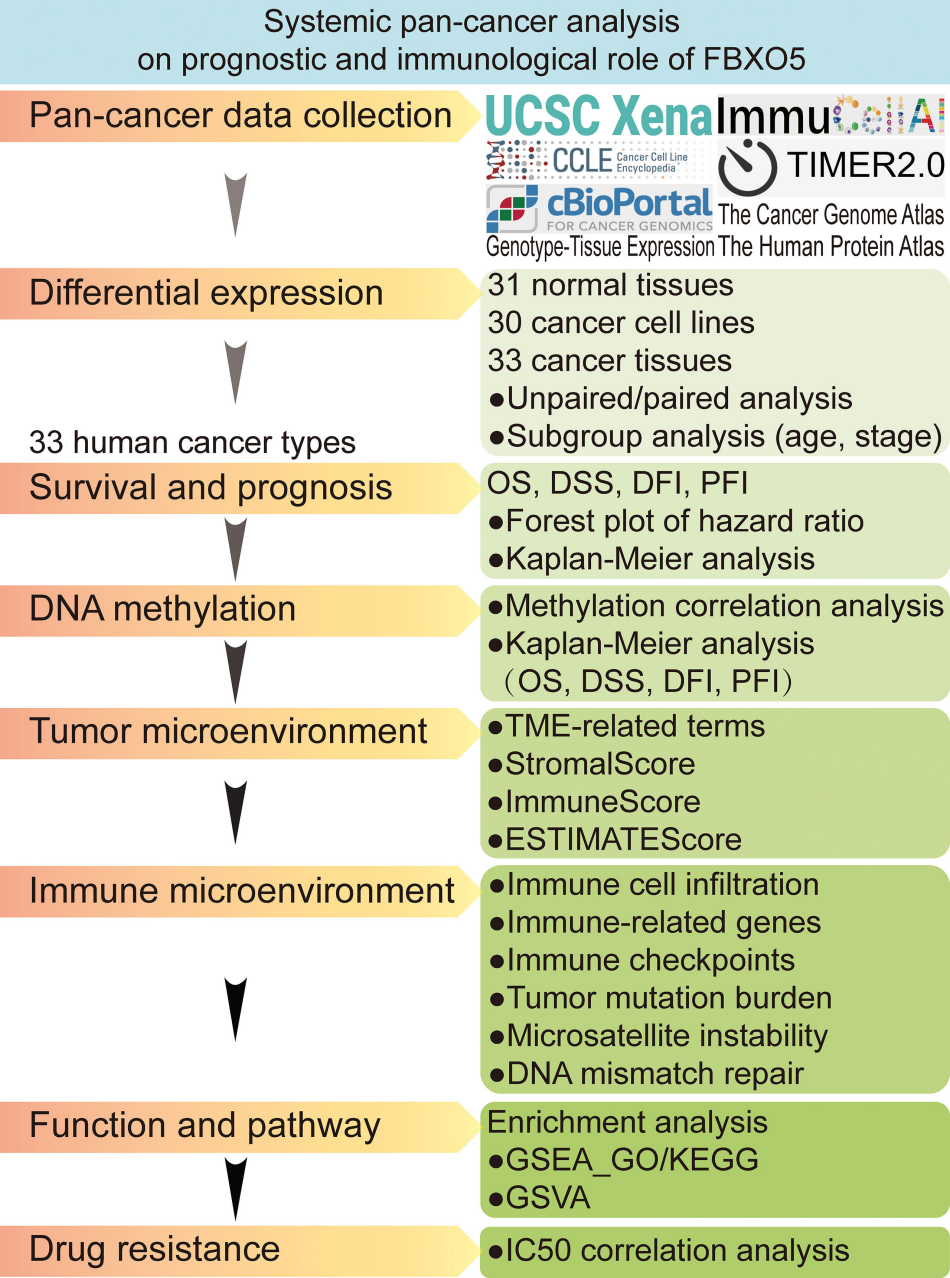
Flow chart of systematic pan-cancer analysis of FBXO5.

In this research, we attempted to conduct a thorough data-mining analysis using multiple public databases to assess the expression and alteration of FBXO5 and visualize the prognostic profiles of FBXO5 in pan-cancer, as well as analyze its correlations with tumor-infiltrating immune cells along with associated immune indicators. **Figure 1** illustrated the design flow and implementing approaches of this study. This work integrally revealed that FBXO5 influenced the prognosis of cancer patients. Upregulation of FBXO5 expression was detrimental to survival in most cancers, with inconsistent findings in only a few types of tumors. Furthermore, the potential biological effects were likely to be linked with DNA methylation, tumor microenvironment, and immune microenvironment. In summary, our findings proposed a comprehensive view of the oncogenic role of FBXO5 in multiple kinds of cancers and suggested that FBXO5 might function as a viable indicator for predicting clinical prognosis and immune therapy response in cancer patients.

## Material and Methods

### Collection of Pan-Cancer Data and Analysis of Gene Differential Expression

FBXO5 gene expression pattern and clinical data in pan-cancer and corresponding normal tissues obtained from the Cancer Genome Atlas (TCGA, https://portal.gdc.cancer.gov) and Genotype-Tissue Expression (GTEx, https://www.gtexportal.org) were analyzed using UCSC Xena (https://xena.ucsc.edu), an online tool for exploring gene expression and processing clinical and phenotypic information. The Cancer Cell Line Encyclopedia (CCLE, https://portals.broadinstitute.org/ccle) database was employed for the purpose of acquiring tumor cell line-related data. Differential expression of FBXO5 in 33 distinct kinds of cancers in contrast with normal samples was explored by means of merging normal tissue data accessed from both the GTEx and TCGA databases. R language software and publicly available R-package “*ggplot2*” were applied to evaluate the differential expression levels by drawing box plots. All expression data preprocessing and normalization were conducted by log_2_(transcripts per million (TPM)+0.001) or log_2_(TPM+1) transformation. Full names and corresponding abbreviations of the 33 types of tumors were listed in the section of Abbreviations.

### Survival and Prognostic Analysis of FBXO5

The survival information and clinical phenotype data concerning each sample were acquired from TCGA database. A total of four survival prognosis indexes, namely overall survival (OS), disease-specific survival (DSS), progression-free interval (PFI), and disease-free interval (DFI), were used to investigate the correlation of FBXO5 expression with the prognosis of cancer patients. The R-packages “*survival*” and “*forestplot*” were employed to conduct a univariate Cox analysis. The median levels of FBXO5 expression were recognized as the expression threshold for classifying the low- and high-expression subgroups. Subsequently, the curves of Kaplan-Meier survival were established utilizing the R-packages “*survminer*” and “*survival*”. The log-rank test was employed to determine statistically significant differences.

### Immunohistochemistry Analysis of FBXO5

The expression pattern of FBXO5 at the protein levels was examined by means of the Human Protein Atlas (HPA, http://www.proteinatlas.org) database containing the protein data of tumor and normal clinical samples. Immunohistochemistry (IHC) photomicrographs of FBXO5 in different types of tumor tissues and corresponding normal control were collected from the HPA. Specifically, the immunohistochemical results based on the antibody against FBXO5 (Cat No. HPA029048; Atlas Antibodies, Sigma-Aldrich) in various normal tissues were downloaded from the tissue section of HPA database, while these data in different tumor tissues were obtained from the pathology section. All sample data are independent of each other. FBXO5 protein staining intensity in cancer tissues was quantified as fold of their respective normal control using Image-Pro Plus 6.0 (Media Cybernetics, USA). According to the statistical differences of these data generated from unpaired two-tailed Student’s *t*-test, representative FBXO5 protein staining pictures were displayed.

### DNA Methylation Analysis of FBXO5

To assess the association between the expression of FBXO5 and DNA methylation in each cancer type involved in this study, we analyzed HM450 methylation data acquired from the cBioPortal database (http://www.cbioportal.org). The correlation between the levels of FBXO5 expression and gene promoter methylation levels was examined and visualized through the R-package “*ggpubr*” for each malignancy studied. Further, the correlation analysis between FBXO5 methylation and tumor prognostic value was also evaluated according to the determination of OS, DSS, DFI, and PFI through applying the survival R-packages to construct the Kaplan-Meier curves.

### Relevance amongst FBXO5 Expression and Tumor Microenvironment

Numerous research reports have demonstrated that tumor microenvironment (TME) performs an integral function in multidrug resistance and tumorigenesis and metastasis (3, 4). To establish the connection between TME and FBXO5 expression, a previously reported method developed by Zeng et al. was applied to estimate the related effects of FBXO5 within TME of 33 cancers (14). Visualization of the associations between FBXO5 expression and TME indicators such as stromal- and immune-relevant signatures was performed in a heatmap using R-based packages.

In addition, ImmuneScore, StromalScore, and ESTIMATEScore for the 33 types of cancers studied were calculated by the algorithm of ESTIMATE (Estimation of Stromal and Immune Cells in Malignant Tumor Tissues Using Expression Data). Increased scores computed in ImmuneScore or StromalScore were deemed to be favorably correlated with the elevated ratio of immunity or stroma, which signified a greater proportion of the corresponding components in TME. Besides, ESTIMATEScore was described as the sum of ImmuneScore and StromalScore, which denoted the combined percentage of both constituents in TME. In this assessment, ImmuneScore and StromalScore of various tumors were subjected to estimation utilizing the R-package “*estimate*” and Pearson’s correlation test.

### Immune Infiltration Analysis of FBXO5

A total of three methods were employed for the purpose of investigating the abundance of infiltrating immune cells in diverse cancers, such as neutrophils, dendritic cells (DCs), CD4^+^ and CD8^+^ T cells, natural killer (NK) cells, mast cells, macrophages, monocytes, and B cells. The first method investigated the associations between the levels of FBXO5 expression and the extent of 24 infiltrating immune cells in 32 distinct cancers except for LAML without immune infiltration data by using the R-packages “*ggplot2*”, “*ggpubr*”, and “*ggExtra*” and the tool CIBERSORT to estimate immune infiltration data from the ImmuCellAI database (http://bioinfo.life.hust.edu.cn/ImmuCellAI#!/). Besides, the TIMER2.0 database (Tumor Immune Estimation Resource, http://timer.comp-genomics.org/) was employed as the auxiliary technique in order to assess the correlations between FBXO5 expression and the levels of tumor-infiltrating immune cells. The third method involved the use of R-packages “*limma*”, “*reshape2*”, and “*RColorBreyer*” for the aim of identifying the relevance between FBXO5 expression and immune-associated genes, such as immune-activating genes, immunosuppressive genes, chemokine genes, chemokine-receptor genes as well as major histocompatibility complex (MHC) genes.

### Correlations Between FBXO5 Expression and Immune Checkpoints, Tumor Mutation Burden, Microsatellite Instability, and DNA Mismatch Repair

The investigation of relationships between FBXO5 and the recognized immune checkpoint genes such as CTLA4, CD274, TIGIT, PDCD1, and LAG3 was conducted in accordance with the database of TCGA. Tumor mutation burden (TMB) is a quantitative biological marker of immune response that reflects the proportion of somatic mutations present in tumor cells (15). The somatic mutation data of the 33 tumors involved in this study were acquired from the UCSC Xena repository (https://tcga.xenahubs.net) for the calculation of TMB scores using a Perl script with the correction by dividing based upon the total exon length. Microsatellite instability (MSI) as a result of DNA mismatch repair deficiency is related with patient outcomes (16, 17), and the MSI data were acquired according to a previously published report (18). Both TMB and MSI are associated with the effectiveness of immunotherapy across diverse cancers. The correlation between FBXO5 expression and TMB or MSI was explored by means of Pearson correlation coefficient, with these findings displayed in the form of radar plots. Mismatch repair (MMR) is an intracellular DNA repair mechanism. Downregulation or functional defects of MMR genes such as MSH2, MSH6, PMS2, MLH1, and EPCAM can lead to irreparable DNA replication errors, resulting in high-frequency somatic mutations and thereby increasing susceptibility to cancer (19). The correlation between FBXO5 and MMR gene expression was determined based upon gene expression profile data from the TCGA cohort and visualized as a heatmap using the R-packages “*reshape2*” and “*RColorBrewer*”.

### FBXO5-Associated Enrichment Analysis in Pan-Cancer

An investigation into the biological effects of FBXO5 in the human cancers studied was conducted by means of Gene Set Enrichment Analysis (GSEA) and Gene Set Variation Analysis (GSVA). The R-packages “*ClusterProfiler*”, “*limma*”, and “*enrichplot*” were employed for the enrichment analyses of Gene Ontology (GO) and Kyoto Encyclopedia of Genes and Genomes (KEGG). GO and KEGG gene sets were obtained from the GSEA website (https://www.gsea-msigdb.org/gsea/index.jsp). GSVA gene set was downloaded from the module “hallmark gene sets” in the Molecular Signatures Database (MSigDB, https://www.gsea-msigdb.org/gsea/msigdb/index.jsp). Moreover, GSVA scores were measured as the correlations between 50 well-defined biological processes or states and FBXO5 expression levels for all tumors.

### Drug Resistance Analysis of FBXO5

For exploring the correlation of FBXO5 expression with drug resistance or sensitivity of tumor cells, the information of various compounds and the corresponding IC50 values and FBXO5 expression data in cancer cell lines were obtained from the GDSC2 dataset (Genomics of Drug Sensitivity in Cancer, https://www.cancerrxgene.org). IC50 here refers to the half maximal inhibitory concentration, which represents the concentration of an inhibitor that is required for 50% inhibition of tumor cell survival. IC50 reflects the tolerance of cells to drugs, i.e., the lower the IC50 value, the more sensitive the cells are to drugs. The correlation between IC50 value of each compound and the levels of FBXO5 expression in cancer cells was analyzed using Spearman correlation coefficient and shown in **Table S1**.

### Statistical Analysis

Statistical data analyses were carried out with the help of R software (https://www.r-project.org/). Comparison of differences between two groups was conducted using Student’s *t*-test or Wilcoxon rank sum test. One-way analysis of variance (ANOVA) was used to compare more than two experimental groups. All survival analyses were performed by applying the Kaplan-Meier product-limit method with a log-rank test and Cox proportional hazards regression model. The correlation between two variables was assessed utilizing Pearson product-moment correlation coefficient. For all statistical differences, *p*-values of less than 0.05, 0.01, 0.001, and 0.0001 were judged to be statistically significant and presented as “*”, “**”, “***”, and “****”, respectively. Besides, “ns” indicated no significance.

## Results

### Differential Expression of FBXO5 Between Tumor and Normal Samples

**Figure 2:**
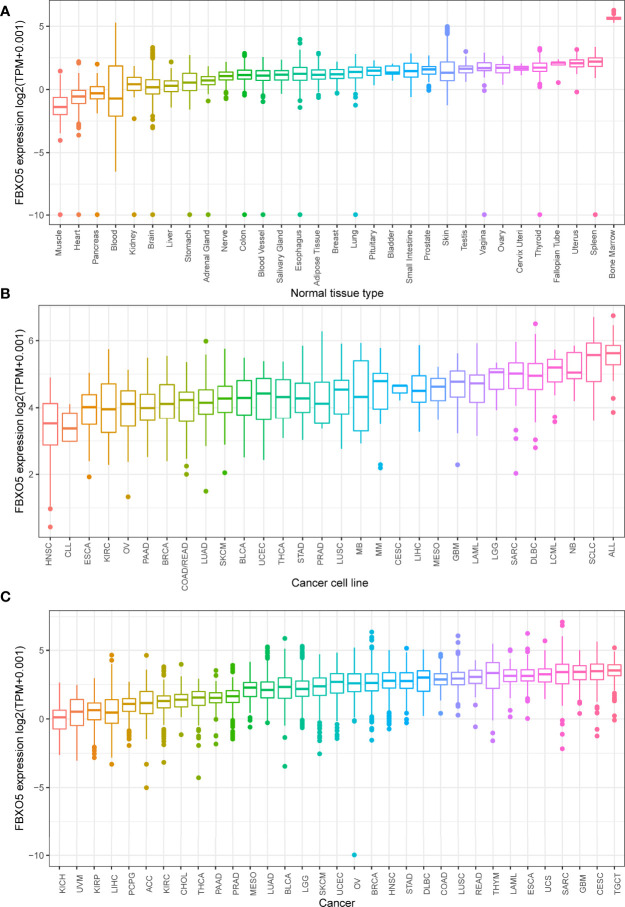
Comparison of FBXO5 expression levels in pan-cancer. **(A)** FBXO5 expression pattern in 31 forms of normal samples from GTEx database. **(B)** FBXO5 expression pattern in 30 kinds of tumor cell lines from CCLE database. **(C)** FBXO5 expression profile in 33 types of cancers from TCGA database.

The physiologic gene expression profiles of FBXO5 among various normal tissues were first analyzed and ranked from low to high, using the GTEx and TCGA data sets (**Figure 2A**). FBXO5 exhibited the highest expression level in bone marrow, but, in general, a majority of other normal samples were found to exhibit low levels of FBXO5 expression. Next, comparative gene expression profiles of FBXO5 among different tumor cell lines that had been obtained from the CCLE database were described in **Figure 2B**, which showed that FBXO5 expression levels were generally higher in up to 30 types of cancer cell lines. Besides, FBXO5 expression levels in various tumors were assayed by the TCGA database, and the findings illustrated that from the 33 cancer tissues analyzed, FBXO5 was expressed with the lowest expression in KICH and with the greatest expression in TGCT (**Figure 2C**).

**Figure 3:**
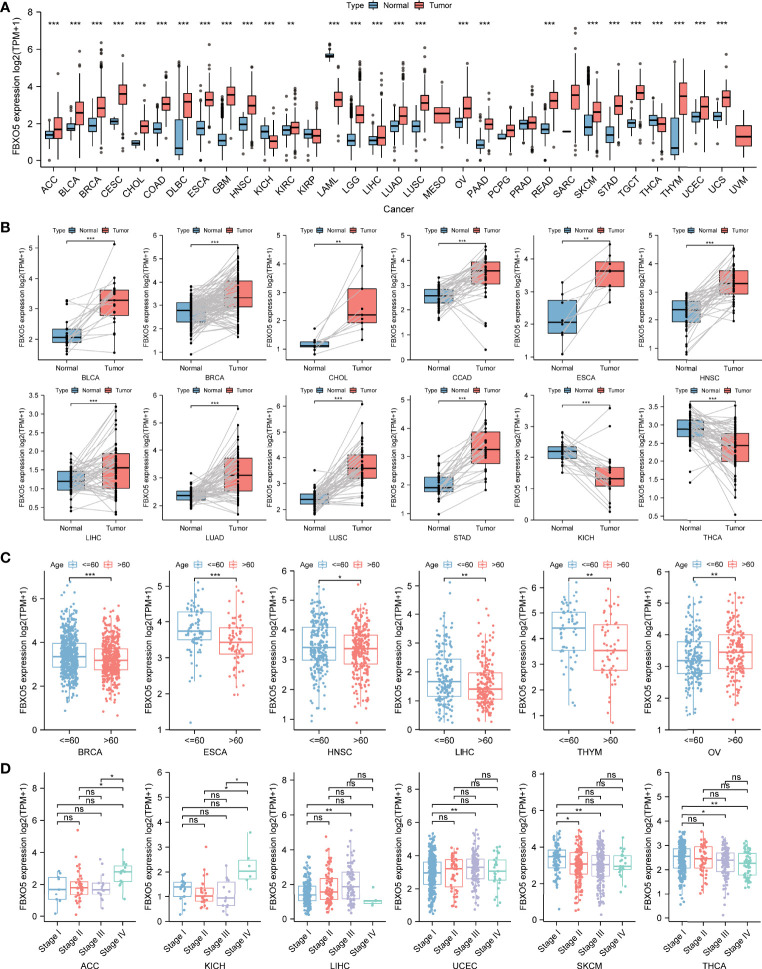
Differential analysis of FBXO5 expression in different kinds of tumors. **(A)** FBXO5 expression differences in tumor tissues from TCGA database compared with normal tissues from GTEx and TCGA databases. **(B)** Paired differential analysis of FBXO5 expression in matched tumor and normal samples from TCGA. **(C)** Differential analysis of FBXO5 expression based on cancer patient age in TCGA. **(D)** Differential analysis of FBXO5 expression based on tumor pathological stages in TCGA. * *p*-value < 0.05, ** *p*-value < 0.01, and *** *p*-value < 0.001. “ns” indicated no significance.

Moreover, differential expression levels of FBXO5 across tumor and normal samples were computed with the aid of the TCGA database (**Figure 3A**). Apart from those tumors with no available normal tissue data including MESO and UVM, statistical significance of FBXO5 expression differences between normal and tumor samples was detected in 27 types of cancers. Among these, highly expressed FBXO5 was further observed in 24 types of cancers, namely ACC, BLCA, BRCA, CESC, CHOL, COAD, DLBC, ESCA, GBM, HNSC, KIRC, LGG, LIHC, LUAD, LUSC, OV, PAAD, READ, SKCM, STAD, TGCT, THYM, UCEC, and UCS (*p-*value < 0.001 in the above tumors except *p-*value < 0.01 in KIRC). Notably, the fold change of upregulation of FBXO5 expression levels among tumor tissues was the highest in GBM compared with the corresponding normal tissues. On the contrary, FBXO5 expression levels showed significantly downregulated in KICH, LAML, and THCA in comparison to their respective normal control (*p-*value < 0.001). Besides, the expression levels of FBXO5 presented no significant differences in KIRP, PCPG, PRAD, and SARC tissues relative to the corresponding normal tissues.

Additionally, the paired differential expression analysis of FBXO5 among tumor and normal tissues was performed followed by a paired Student’s *t*-test. Increased expression of FBXO5 in BLCA, BRCA, CHOL, COAD, ESCA, HNSC, LIHC, LUAD, LUSC, and STAD while decreased expression in KICH and THCA was respectively confirmed in comparison of matched normal samples (**Figure 3B**).

Next, the differential analysis of FBXO5 expression in each tumor type were examined in accordance with the age of patients, which suggested that patients aged > 60 years old had lower expression levels of FBXO5 compared with those aged ≤ 60 years old in several tumor types including BRCA, ESCA, HNSC, LIHC, and THYM, while patients aged > 60 years old with OV had higher FBXO5 expression than those aged ≤ 60 years old (**Figure 3C**). Moreover, the comparison of FBXO5 expression across different pathological stages of each cancer type was assessed, which indicated that FBXO5 expression was higher in more advanced stages of the four malignancies, namely ACC, KICH, LIHC, and UCEC. However, the expression of FBXO5 in higher stages appeared lower in SKCM and THCA (**Figure 3D**).

Subsequently, we further explored the protein expression levels of FBXO5 across tumor and normal clinical samples based upon the HPA database, as depicted in **Supplementary Figures 1A–J**. Quantitative analysis of IHC showed that FBXO5 protein staining intensity in BRCA, CESC, COAD, LIHC, OV, PAAD, STAD, TGCT, and UCEC tissues was respectively detected to be more obvious than weak FBXO5 staining of normal breast, cervix, liver, ovaries, pancreas, and stomach tissues, and also stronger compared with moderate FBXO5 staining of normal colon, testes, and endometrium tissues (**Supplementary Figure 1K**). In contrast, FBXO5 IHC staining in THCA was lighter than that in normal thyroid gland samples (**Supplementary Figure 1K**). Therefore, these IHC staining data were consistent with the sequencing results of FBXO5 at the transcriptome level.

### Pan-Cancer Prognostic Value of FBXO5

**Figure 4:**
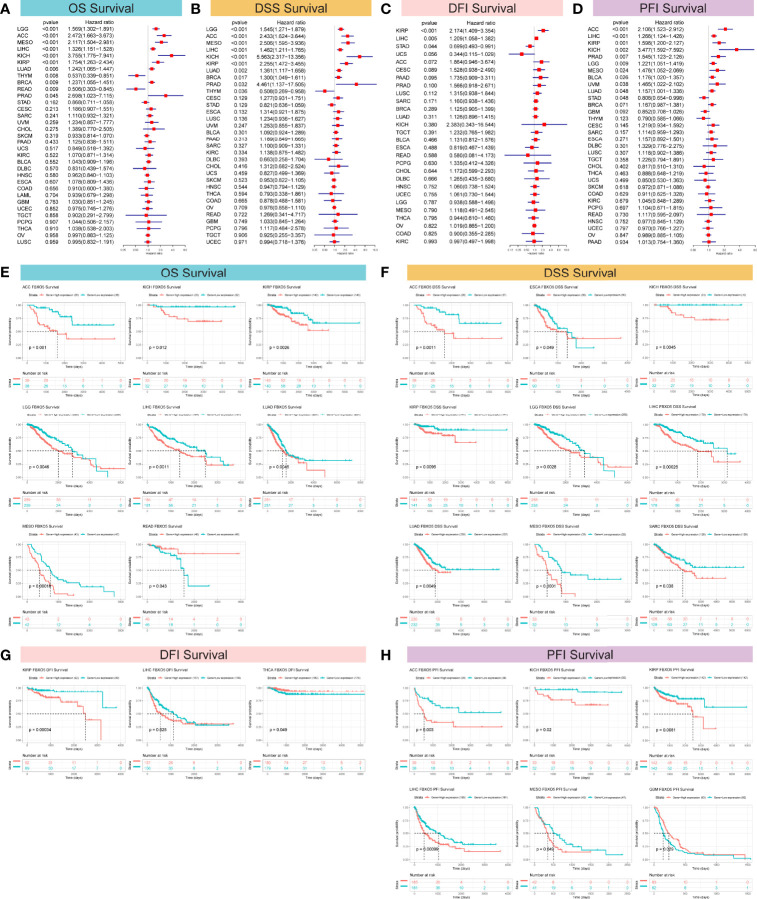
Correlation of FBXO5 expression with survival prognosis in the distinct malignancies studied. **(A–D)** Forest plots revealing the associations of FBXO5 with OS **(A)**, DSS **(B)**, DFI **(C)**, and PFI **(D)** in the indicated tumors, respectively. **(E–H)** Kaplan-Meier curves showing the relationships of FBXO5 expression with OS **(E)**, DSS **(F)**, DFI **(G)**, and PFI **(H)** in the indicated cancers, respectively.

For the purpose of clarifying the correlation between FBXO5 expression and tumor prognosis, hazard ratio statistics for OS, DSS, DFI, and PFI were processed *via* forest plots for each cancer included in this study. According to the univariate Cox regression analysis, FBXO5 was a remarkable risk factor for OS in LGG, ACC, MESO, LIHC, KICH, KIRP, LUAD, and BRCA (*p*-value < 0.001 except LUAD and BRCA in which *p*-value < 0.01), while a protective factor in THYM (*p*-value = 0.008) and READ (*p*-value = 0.009) (**Figure 4A**). Next, Cox regression analysis of DSS identified that FBXO5 was a prominent risk factor in LGG, ACC, MESO, LIHC, KICH, KIRP, and LUAD (*p*-value < 0.001 other than LUAD in which *p*-value = 0.002). On the other side, FBXO5 served as a protective factor in THYM (*p*-value = 0.036), as displayed in **Figure 4B**. Subsequently, DFI Cox regression analysis regarded FBXO5 as a risk factor in HNSC (*p-*value < 0.001) and LIHC (*p-*value = 0.005) but a protective factor in STAD (*p-*value = 0.044), as depicted in **Figure 4C**. Furthermore, Cox regression analysis of PFI revealed that FBXO5 acted as an unfavorable factor for patients with ACC, LIHC, KIRP, KICH, PRAD, and LGG (*p*-value < 0.001 for ACC and LIHC, *p*-value = 0.001 for KIRP, and *p*-value < 0.01 for KICH, PRAD, and LGG), whereas a protective factor in STAD (*p*-value < 0.05), as shown in **Figure 4D**.

Correspondingly, Kaplan-Meier survival analyses of OS, DSS, DFI, and PFI were further investigated in the 33 forms of cancers studied. Kaplan-Meier survival curves for OS showed that elevated FBXO5 expression levels were evidently correlated with an unfavorable prognosis of cancer patients suffering from ACC, KICH, KIRP, LGG, LIHC, LUAD, and MESO (*p*-value < 0.01 other than KICH in which *p*-value < 0.05), whereas significantly correlated with longer survival time in patients with READ (*p-*value = 0.043) (**Figure 4E**). Meanwhile, Kaplan-Meier DSS survival analysis demonstrated a remarkable relationship of high levels of FBXO5 expression and poor survival outcomes in patients having ACC, ESCA, KICH, KIRP, LGG, LIHC, LUAD, MESO, and SARC (*p-*value < 0.01 other than ESCA and SARC in which *p*-value < 0.05) (**Figure 4F**). Then, Kaplan-Meier survival analysis of DFI revealed a significant connection between high FBXO5 expression and poor prognosis in KIRP (*p-*value = 0.00034) and LIHC (*p-*value = 0.025). Nevertheless, increased expression of FBXO5 exhibited a protective effect on THCA patient outcomes (*p-*value = 0.049) (**Figure 4G**). Moreover, Kaplan-Meier PFI analysis showed that patients with ACC, KICH, KIRP, LIHC, and MESO had relatively longer survival time probably because of low expression levels of FBXO5 (*p-*value < 0.01 other than KICH and MESO in which *p*-value < 0.05). However, contrasting results were observed in PFI survival of patients with GBM (*p-*value = 0.029) (**Figure 4H**). Taken together, these results implied that high FBXO5 expression generally contributed to unfavorable patient prognosis and survival in a variety of tumor types.

### Correlativity of FBXO5 Expression and DNA Methylation Modification

**Figure 5:**
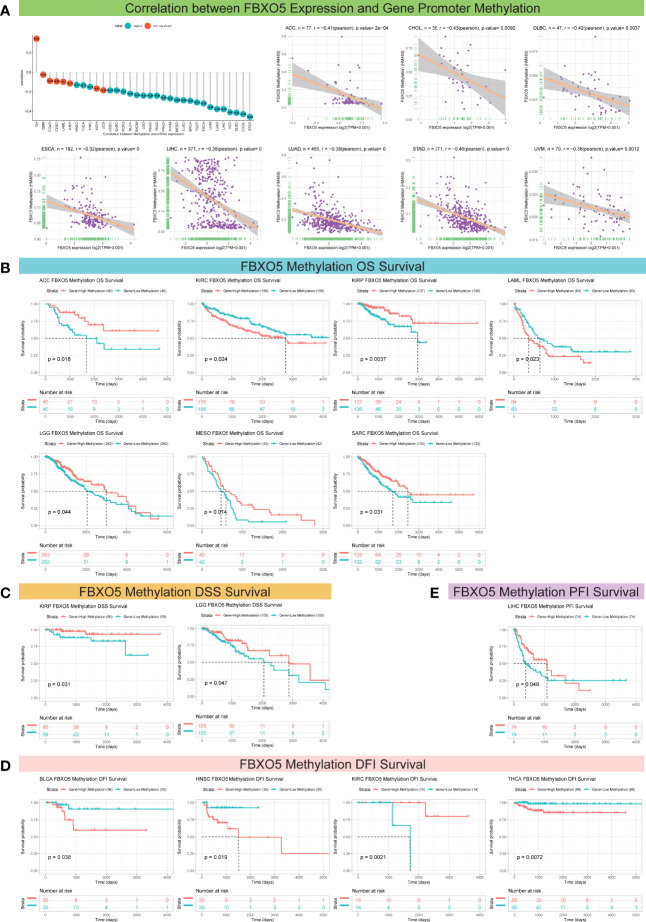
Association between FBXO5 expression and gene promoter methylation in pan-cancer. **(A)** Lollipop plot and Pearson’s analyses depicting the correlation between FBXO5 expression and DNA methylation in the indicated tumors. **(B–E)** Kaplan-Meier curves illustrating the relationships between FBXO5 methylation levels with OS **(B)**, DSS **(C)**, DFI **(D)**, and PFI **(E)** in the indicated cancers, respectively.

Next, FBXO5 promoter methylation levels accompanied by changes in FBXO5 expression were estimated using the cBioPortal database and the results reflected significant correlations between FBXO5 expression and methylation in a total of 25 tumors as indicated by the lollipop chart in **Figure 5A**. Among these 25 cancer types, FBXO5 expression all showed Pearson’s negative correlations with gene promoter methylation levels, and the former eight malignancies with the greatest inverse association were respectively presented in **Figure 5A**, including STAD, CHOL, DLBC, ACC, LIHC, LUAD, UVM, and ESCA (*p*-value < 0.001 except CHOL, DLBC, and UVM in which *p*-value < 0.01). Pearson’s correlation analyses of the other different cancers were displayed in **Supplementary Figure 2**.

On the other hand, for the aim of exploring the correlation between FBXO5 promoter methylation and survival prognosis (OS, DSS, DFI, and PFI), Kaplan-Meier analyses were performed for the 33 forms of cancers studied. Enhanced FBXO5 methylation was demonstrated to be a protective indicator for superior OS in patients diagnosed with ACC, KIRP, LGG, MESO, and SARC (*p*-value < 0.05 except KIRP in which *p*-value < 0.01), whereas a deleterious indicator that was prone to result in low survival probability of KIRC (*p*-value = 0.024) and LAML (*p*-value = 0.023) (**Figure 5B**). DSS analysis demonstrated that FBXO5 methylation functioned as a protective marker in patients experiencing KIRP (*p*-value = 0.031) and LGG (*p*-value = 0.047) (**Figure 5C**). Besides, FBXO5 methylation levels exhibited a significant positive correlation with DFI survival in patients experiencing KIRC (*p*-value = 0.0021) although FBXO5 methylation acted as a harmful factor in patients with BLCA (*p*-value = 0.038), HNSC (*p*-value = 0.019), and THCA (*p*-value = 0.0072) (**Figure 5D**). Moreover, as far as PFI be concerned, a lower FBXO5 methylation level was overtly relevant with a poorer prognosis in LIHC patients (*p*-value = 0.048) (**Figure 5E**).

### Association Between FBXO5 Expression and Tumor Microenvironment

**Figure 6:**
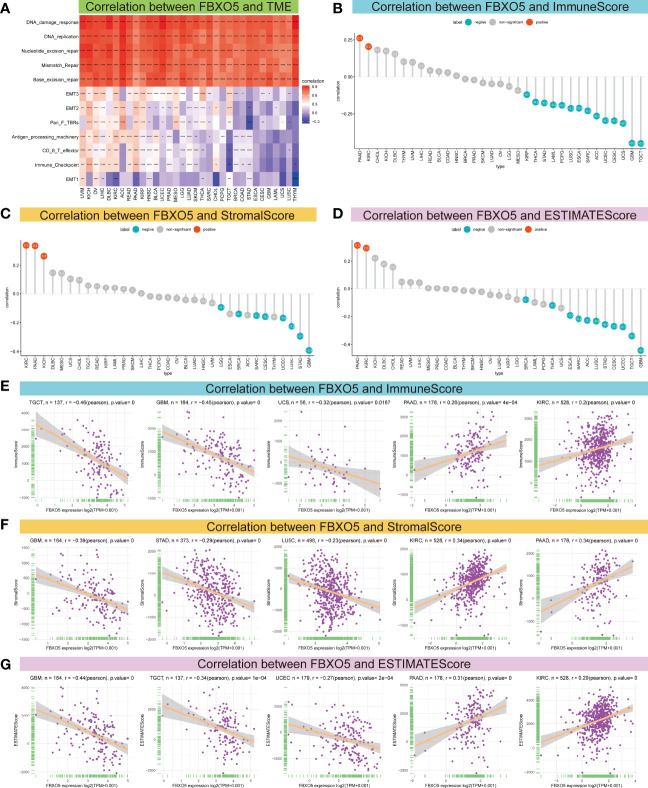
Association between FBXO5 expression and TME. **(A)** Heatmap presenting the correlation strength between FBXO5 expression and TME-related terms in pan-cancer. * *p*-value < 0.05, ** *p*-value < 0.01, *** *p*-value < 0.001, and **** *p*-value < 0.0001. **(B–D)** Lollipop plots displaying the correlations of FBXO5 expression with ImmuneScore **(B)**, StromalScore **(C)**, and ESTIMATEScore **(D)** in pan-cancer, respectively. **(E-G)** Pearson’s analyses of the relationships between FBXO5 expression and ImmuneScore **(E)**, StromalScore **(F)**, and ESTIMATEScore **(G)** in the indicated tumor types, respectively.

Emerging researches have established that TME exerts a pivotal function during the onset and progression of tumors (3, 4). Therefore, it is essential to examine the correlation between TME and FBXO5 expression levels. **Figure 6A** described a heatmap of the correlation strength between FBXO5 expression and TME terms, showing that DNA damage response, DNA replication, nucleotide excision repair, mismatch repair as well as base excision repair were highly positively connected to the expression of FBXO5 in the indicated cancers. Subsequently, the ESTIMATE algorithm was implemented to compute the ImmuneScore, StromalScore, and ESTIMATEScore in the 33 distinct malignancies and analyze the Pearson’s correlations between the expression of FBXO5 and the above three scores in pan-cancer. The lollipop plots presented an in-depth understanding of FBXO5 expression and TME scores in different cancers (**Figures 6B–D**). In PAAD and KIRC, the findings indicated that FBXO5 expression exhibited significant positive correlations with the ImmuneScore (**Figure 6B**), StromalScore (**Figure 6C**), and ESTIMATEScore (**Figure 6D**), respectively. Conversely, notable inverse correlations were discovered between FBXO5 expression and all three scores of SARC, CESC, UCEC, LUSC, STAD, and GBM (**Figures 6B-D**). Furthermore, regarding the value of Pearson’s r, the top three cancers with predominant negative correlations and the top two cancers with remarkable positive correlations of FBXO5 expression and TME-relevant scores included TGCT, GBM, UCS, PAAD, and KIRC (**Figure 6E**, sorted by ImmuneScore); GBM, STAD, LUSC, KIRC, and PAAD (**Figure 6F**, sorted by StromalScore); GBM, TGCT, UCEC, PAAD, and KIRC (**Figure 6G**, sorted by ESTIMATEScore), respectively. In addition, the Pearson’s correlations of FBXO5 expression levels with ImmuneScore, StromalScore, and ESTIMATEScore of the other malignancies investigated were separately displayed in **Supplementary Figures 3**, **4** and **5**.

### Correlation of FBXO5 Expression With Pan-Cancer Immune Cell Infiltration

**Figure 7:**
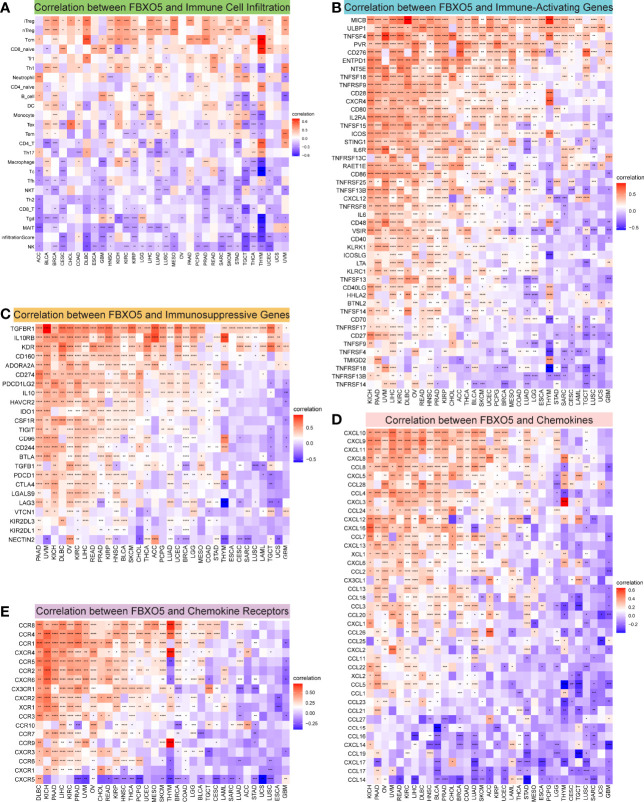
Correlation between FBXO5 expression and tumor immunity. **(A)** Heatmap showing the association between FBXO5 expression and the infiltration of immune cells in pan-cancer generated by using the ImmuCellAI database. **(B–E)** Heatmaps indicating the co-expression of FBXO5 with immune-relevant genes in pan-cancer, including immune activation genes **(B)**, immunosuppressive genes **(C)**, chemokine genes **(D)**, and chemokine-receptor genes **(E)**. * *p*-value < 0.05, ** *p*-value < 0.01, *** *p*-value < 0.001, and **** *p*-value < 0.0001.

Growing researches have suggested that tumor-infiltrating immunocytes could have a critical impact on the survival status of patients (20). From this point of view, the correlations between the expression levels of FBXO5 and the infiltration abundance of 24 distinct immune cell subtypes were analyzed at a pan-cancer level using the ImmueCellAI database. It was revealed that immune cell infiltration degree was strongly related to FBXO5 expression in most cancers (**Figure 7A**). For instance, the expression of FBXO5 exhibited a remarkable positive correlation with regulatory T (Treg) cells and central memory T (Tcm) cells. In comparison, the FBXO5 expression exhibited a substantial negative correlation with natural killer (NK) cells, CD8^+^ T cells, and T-helper 2 (Th2) cells. Interestingly, various relationships between FBXO5 expression and distinct T cell subsets were further discovered, as illustrated in **Supplementary Figure 6A**. For example, the expression of FBXO5 presented an inverse association with the infiltrating levels of CD4^+^ Tcm cells, CD4^+^ effector memory T (Tem) cells, and natural killer T (NKT) cells in most tumors based upon the XCELL algorithm. Besides, a close positive correlation between FBXO5 expression and infiltrating macrophages was observed in PRAD (*p*-value < 0.0001), whereas a notable negative correlation was identified in GBM, THCA, and THYM (*p*-value < 0.0001 for the three cancers) (**Figure 7A**). By use of the TIMER2.0 database, similar results about FBXO5 expression-related macrophage infiltration were also found in PRAD based upon the TIMER algorithm, and GBM, THCA, and THYM based upon the XCELL algorithm (**Supplementary Figure 6A**).

Afterwards, the correlations of expression levels between FBXO5 and immune-related genes that encode immune-activating, immunosuppressive, chemokine, chemokine-receptor proteins as well as MHC were investigated across cancers (**Figures 7B–E** and **Supplementary Figure 6B**). The heatmaps demonstrated that FBXO5 exhibited a significant co-expression relationship with most immune activation and immunosuppressive genes in various cancers, particularly in KICH, PAAD, UVM, LIHC, KIRC, DLBC, OV, READ, HNSC, PRAD, and KIRP (**Figures 7B, C** **)**. Furthermore, both chemokine and chemokine-receptor genes were strongly co-expressed with FBXO5 in pan-cancer (**Figures 7D, E** **)**. For example, in KICH and PAAD, there was a positive connection of the expression levels of FBXO5 with most chemokine and chemokine-receptor genes. Concurrently, **Supplementary Figure 6B** elucidated that FBXO5 expression was positively associated with almost all MHC genes in KIRC, UVM, PAAD, KICH, and OV, while inversely correlated in THYM, TGCT, and GBM. To conclude, these data inferred that FBXO5 might contribute to regulating immune cell infiltration and the biological functions of various immune-related genes in the tumor immune microenvironment of most tumor types.

### Associations of FBXO5 Expression With Immune Checkpoints, Tumor Mutation Burden, Microsatellite Instability, and DNA Mismatch Repair

Immune checkpoints that are responsible for regulating the degree of immune activation and play a crucial role in autoimmunity and immune surveillance of tumor cells, have already been identified as the inhibitory targets of cancer immunotherapy (5, 6). Subsequently, the correlations were studied between FBXO5 and five major immune checkpoint genes, namely CTLA4, CD274, TIGIT, PDCD1, and LAG3. In most cancers, FBXO5 expression was highly related with the levels of immune checkpoint gene expression (**Supplementary Figure 7**).

**Figure 8:**
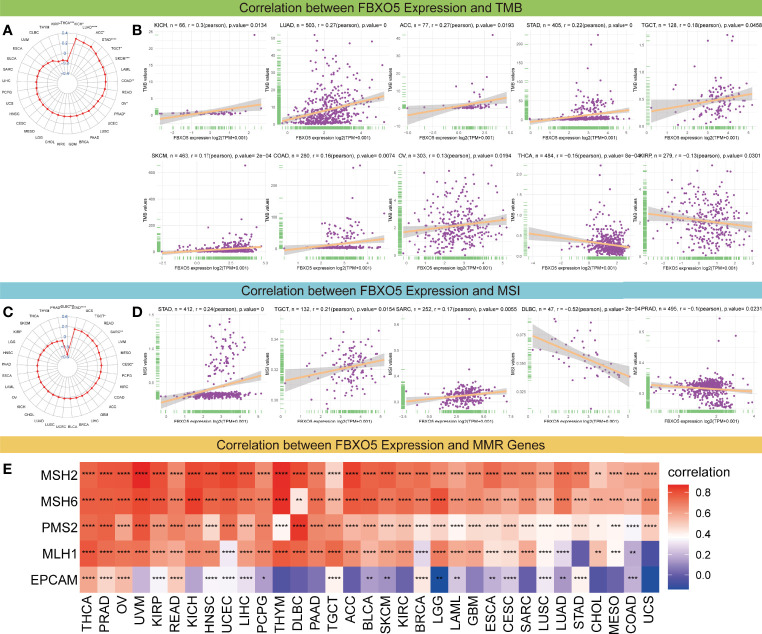
Correlations of FBXO5 expression with TMB, MSI, and MMR. **(A)** Radar map of the association between FBXO5 expression and TMB levels in pan-cancer. The values in blue stand for the range and the lines in red stand for the correlation coefficients. **(B)** Pearson’s correlation between FBXO5 expression and TMB levels in the indicated cancers. **(C)** Radar map of the association between FBXO5 expression and MSI frequencies in pan-cancer. **(D)** Pearson’s correlation between FBXO5 expression and MSI frequencies in the indicated cancers. **(E)** Heatmap illustrating the association between FBXO5 expression and five MMR genes (MSH2, MSH6, PMS2, MLH1, and EPCAM) in pan-cancer. * *p*-value < 0.05, ** *p*-value < 0.01, *** *p*-value < 0.001, and **** *p*-value < 0.0001.

Additionally, considering the essential roles of TMB and MSI in the prediction of the response to immune therapy across cancers, the link between FBXO5 expression and the values of TMB or MSI was also explored. Overall, these two metrics varied remarkably among different cancer types (**Figures 8A, C** **)**. FBXO5 expression showed to be positively relevant to TMB in KICH, LUAD, ACC, STAD, TGCT, SKCM, COAD, and OV (**Figure 8B**). In contrast, FBXO5 expression inversely correlated to TMB in THCA and KIRP (**Figure 8B**). Furthermore, FBXO5 expression had a positive correlation with MSI in STAD, TGCT, and SARC (**Figure 8D**). Nonetheless, FBXO5 expression inversely correlated to MSI in PRAD and DLBC (**Figure 8D**). On balance, the aforementioned results strongly indicated that FBXO5 was well associated with tumor immunity. Thus, FBXO5 might be taken as a viable biomarker for indicating the immunotherapy response in these tumor types.

Furthermore, deficient mismatch repair (dMMR) is an unneglected mechanism of tumorigenesis and development, which suggests that the potential relationship between FBXO5 and MMR needs to be studied in pan-cancer. The results displayed that FBXO5 expression was significantly positively associated with almost each of the five MMR genes (MSH2, MSH6, PMS2, MLH1, and EPCAM) in most tumors (**Figure 8E**). Specially, FBXO5 expression was negatively correlated with EPCAM in LGG (**Figure 8E**). These data indicated that FBXO5 might regulate the tumor progression by mediating the repairment of DNA mismatch across cancers.

### Enrichment Analysis of FBXO5 in Pan-Cancer

**Figure 9:**
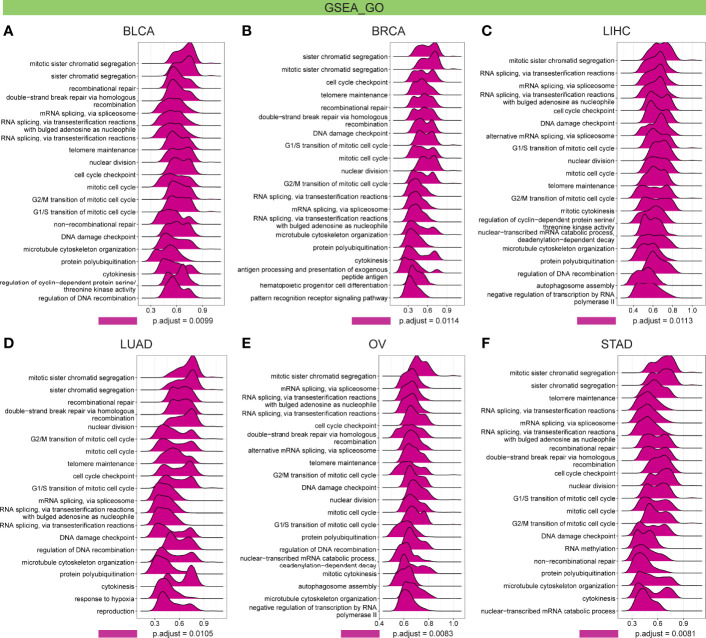
GO annotations of FBXO5 in the indicated six types of tumors using GSEA, including **(A)** BLCA, **(B)** BRCA, **(C)** LIHC, **(D)** LUAD, **(E)** OV, and **(F)** STAD.

**Figure 10:**
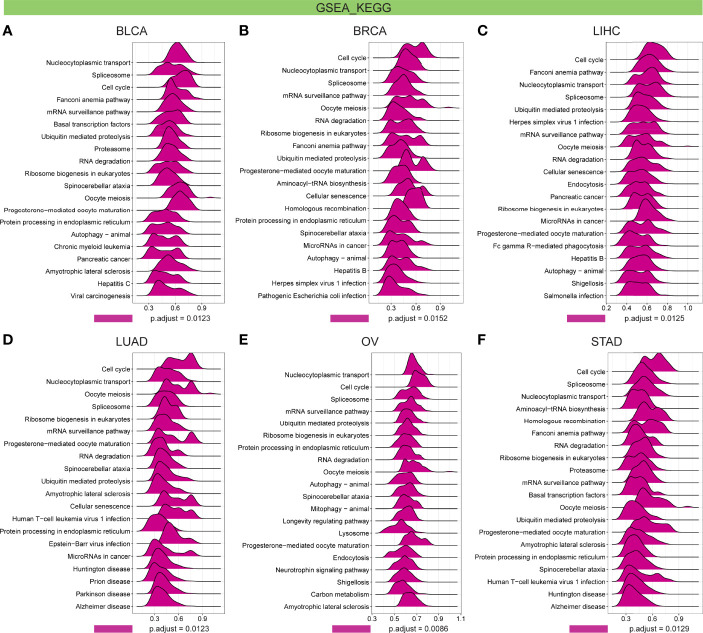
KEGG annotations of FBXO5 in the indicated six types of tumors using GSEA, including **(A)** BLCA, **(B)** BRCA, **(C)** LIHC, **(D)** LUAD, **(E)** OV, and **(F)** STAD.

Further, GSEA and GSVA were carried out to explore the underlying biological relevance of FBXO5 in tumor tissues. GO analysis indicated that FBXO5 was significantly linked to the functions of cell division and cell cycle regulation, including sister chromatid segregation, telomere maintenance, nuclear division, cell cycle checkpoint, RNA splicing, and DNA damage checkpoint in malignancies of various types, such as BLCA, BRCA, LIHC, LUAD, OV, and STAD (**Figures 9A–F**). KEGG-enriched terms revealed that the major associations of FBXO5 with the above six cancers existed in the processes of nucleocytoplasmic transport, cell cycle, cellular senescence, ubiquitin-mediated proteolysis, and microRNAs in cancer (**Figures 10A–F**). Of note, FBXO5 was found to be critically involved in immune-associated tumorigenic virus infectious diseases, including hepatitis B, hepatitis C, human T-cell leukemia virus 1 infection, and Epstein-Barr virus infection (**Figures 10A–F**).

Meanwhile, the GSVA data reinforced that FBXO5 expression was positively correlated with mitotic spindle, G2/M checkpoint, mTORC1 signaling, PI3K/Akt signaling, and protein secretion in the above six malignancies, as well as inflammatory response of cell immune factors in BLCA, BRCA, and LIHC such as interferon-alpha, interferon-gamma, IL6, and TGF-beta, but in general it was inversely associated with K-ras signaling DN, myogenesis, bile acid metabolism, xenobiotic metabolism, and p53 pathway (**Supplementary Figures 8A–F**). These results together implied that FBXO5 played a key role in regulating the tumor occurrence and progression and immune microenvironment.

### Correlation of FBXO5 Expression With Drug Resistance

At present, in clinical practice, multidrug resistance (MDR) of cancer cells is an unneglected link leading to recurrent tumor and affecting prognosis and survival. Hence, for evaluating the potential of FBXO5 in guiding clinical treatment, the correlation between FBXO5 and drug resistance of tumor cells was computed based upon IC50 values of drugs along with FBXO5 expression levels. The results suggested that FBXO5 exhibited a significant positive association with IC50 values of seven compounds but a notable negative correlation with IC50 values of 153 compounds (**Table S1**). Besides, there were no significant relevance between FBXO5 and IC50 in a total of 32 compounds (**Table S1**). Therefore, increased expression of FBXO5 can make tumor cells more sensitive to most kinds of compounds, which implies that the therapies of most compounds become effective in cancer patients with high FBXO5 expression. From the perspective of translational medicine, detection of FBXO5 expression may be used to guide the efficacy prediction of drug clinical treatment in tumors, and also contribute to the accurate selection of antitumor drugs.

## Discussion

APC is well known to be a critical ubiquitin ligase that governs the cell cycle progression through mitosis to G1-phase (21). FBXO5-encoded protein is a significant regulator of APC activity and it functions in cell cycle modulation through effectively stabilizing the ubiquitination substrates of APC after preventing ubiquitin chain elongation (22). Recently, several bioinformatics researches proved that FBXO5 could specifically regulate multiple tumor behaviors and exhibited a crucial role in the tumorigenesis and prognosis of several cancers, including squamous cell lung carcinoma (23), breast cancer (24), hepatocellular carcinoma (25), and HPV-positive cervical cancer (26). Above all, these studies speculated that FBXO5 could be regarded as a potential oncogenic factor or therapeutic target among the above tumor types.

On the premise of existing evidence, the current work performed a thorough investigation for pan-cancer analysis of FBXO5 across malignancies. This study constructed a detailed exploration of FBXO5 expression differences, underlying functions, and prognostic significance in 33 human cancers. Rising FBXO5 expression was confirmed to be remarkably correlated with unfavorable clinical outcomes in a variety of cancers. Further demonstration revealed that abnormal FBXO5 expression in most cancers was significantly correlated with TME, immune infiltration, DNA methylation, immune checkpoints, TMB, MSI, and MMR. Collectively, FBXO5 might confer an instrumental function in indicating tumorigenesis, prognosis, as well as tumor immunity, which is briefly summarized in **Table S2**.

Our data first showed a significant increase of FBXO5 expression in 24 cancers, while decrease in KICH, LAML, and THCA in contrast to the corresponding normal tissues. These findings were in agreement with earlier research reports on ovarian clear cell carcinoma (9), esophageal squamous cell carcinoma (10), squamous cell lung carcinoma (23), breast cancer (11, 24), hepatocellular carcinoma (12, 25), and cervical cancer (26). Meanwhile, FBXO5 expression was analyzed to be correlated with the tumor stage. In patients having UCEC, LIHC, KICH, and ACC, FBXO5 expression was higher at stage III or IV than stage I or II. Hence, the current data provided a hint that FBXO5 could serve as an oncogene in most tumors.

The present research also discovered that FBXO5 expression was related to tumor prognosis, which suggested that the overexpression of FBXO5 was a hazard factor of cancers and could cause a poorer prognosis in terms of OS, DSS, PFI, and DFI. Similarly, existing researches also demonstrated that FBXO5 upregulation was critically correlated to the poor prognosis of esophageal squamous cell carcinoma (10), hepatocellular carcinoma (12), squamous cell lung carcinoma (23), and breast cancer (11, 24). Notably, highly expressed FBXO5 in STAD (stomach adenocarcinoma) was a protective factor for DFI and PFI survival, and this was different from most cancers in which increased FBXO5 acted as an unfavorable prognostic factor, implying that FBXO5 may have specific functions in STAD. A previous study on the role of CRIP1 can well explain the protective role of FBXO5 in gastric cancer (27). Simply put, CRIP1 is overexpressed in gastric cancer and CRIP1 deficiency inhibits the process of homologous recombination and increases susceptibility to chemotherapy in gastric cancer cells. FBXO5 can block CRIP1-promoted homologous recombination repair by preventing nuclear enrichment of RAD51, thereby restoring chemotherapy sensitivity of gastric cancer cells with high CRIP1 expression. Due to the negative correlation between CRIP1 expression and survival time in gastric cancer patients, FBXO5 may exhibit a potential protective role in the prognosis of gastric cancer through inhibiting the identified function of CRIP1 in this report.

DNA methylation is a type of DNA chemical modification that functions as a crucial regulator of gene transcription. Abnormalities of DNA methylation levels perform an indispensable function in the onset and progression of many tumors (28). Pan-cancer survival analysis showed that FBXO5 promoter methylation levels were obviously correlated with better OS in patients diagnosed as ACC, KIRP, LGG, MESO, and SARC, which was opposite in KIRC and LAML patients. These results uncovered that FBXO5 could be developed as a promising predictive biomarker for clinical prognosis of various malignancies.

TME comprising various infiltrating immune and stromal cells confers instrumental functions in the pathogenesis and development of cancer (3, 4) and meanwhile it can also prominently affect therapeutic response and clinical outcomes (29). The findings of the present research elucidated that FBXO5 expression in 33 malignancies positively correlated to TME involving DNA damage and repair. Of note, DNA damage response (DDR) genes are frequently mutated in almost all cancer types (30). Consequently, deficiency of DDR/DNA repair can lead to accumulative somatic mutations and increased susceptibility to cancer (31). This study suggested that FBXO5 might affect DNA damage and repair and induce carcinogenesis. Typically, the function of ESTIMATEScore is its ability to determine the purity of tumor. The higher the ESTIMATEscore, the lower the tumor purity, and a low purity denotes an advanced cancer stage with a poor prognosis (32, 33). In terms of the ESTIMATEscore, a positive correlation with FBXO5 expression was found only in PAAD and KIRC. On the contrary, a negative relationship with FBXO5 expression existed in diverse cancers.

In addition, tumor-infiltrating immunocytes can promote or antagonize the tumorigenesis and progression in a two-way manner (34). In order to truly comprehend the implications of the tumor immune microenvironment, the correlation between FBXO5 expression and the abundance of infiltrating immune cells in the 33 malignancies described was evaluated in the present study, which highlighted that FBXO5 expression showed a positive correlation with tumor-infiltrating Treg and Tcm cells, and contrastingly a negative correlation with the infiltration degree of NK/NKT cells, CD8^+^ T cells, and Th2 cells. Furthermore, there was a positive relationship between FBXO5 expression and infiltrating macrophages in BLCA, KICH, KIRC, LUAD, and PRAD. Thus, FBXO5 was likely to interact with immunocytes across many malignancies, exhibiting a wide range of applicability. Subsequently, the co-expression correlations of FBXO5 and the genes encoding immune activating and suppressive factors, chemokines, chemokine receptors, as well as MHC were examined, and the findings together illustrated that FBXO5 expression was widely correlated with different immune factors and immunocytes infiltrating into tumors.

MSI and TMB are two valuable indexes both having essential connections with the sensitivity of immune checkpoint inhibitors (35–37). Gastroesophageal cancer patients manifested as high-frequency MSI (MSI-H) present an increased response rate and favorable outcomes to immunotherapy (16). Of note, MSI-H in colorectal cancer is proved to be an independent predictor for clinical features and prognosis (17). Moreover, recent clinical studies also revealed that survival prognosis was effectively improved across cancers with high somatic TMB levels following immune checkpoint inhibitor therapy (38, 39). The present data uncovered that FBXO5 expression had a general relationship with multiple immune checkpoint genes and MMR genes in most cancers. Further, FBXO5 expression was also significantly correlated with MSI or TMB in multiple cancer types. Summarily, the results mentioned above suggested that aberrant FBXO5 expression affected the values of TMB and MSI, thus impacting the treatment effects on patients receiving immunotherapy. Hence, FBXO5 could become a potential immunotherapeutic target for tumors.

Regarding possible regulatory mechanisms, both GO and KEGG enrichment analyses indicated that FBXO5 was closely related to the functions of cell division and cell cycle regulation. Notably, FBXO5 was reported to promote the proliferation of breast cancer cells through PI3K/Akt signaling pathway, leading to a grim prognosis, whereas PI3K inhibitor LY294002 repressed FBXO5 expression and cell proliferative capacity (11). Interestingly, in mammalian cells, FBXO5 initiated the progress of cell cycle *via* the mechanism of converting from an APC/C^CDH1^ substrate to an APC/C^CDH1^ inhibitor (40). In line with previous studies (9–13, 23–26), GSEA and GSVA analyses obtained herein demonstrated that FBXO5 might influence the pathogenesis or immunity of cancer by participating in the processes of DNA damage checkpoint, cellular senescence, inflammatory response, PI3K/Akt/mTOR signaling pathway, and p53 signaling pathway. In brief, these results offered a theoretical basis for interpreting the oncogenic role and immunological function of FBXO5 in pan-cancer.

However, the current study has several limitations. First, systematic bias exists due to multiple information sources retrieved from different databases for analysis. Second, there are only a few molecular studies on FBXO5 in cancers, including ovarian clear cell carcinoma (9), esophageal squamous cell carcinoma (10), breast carcinoma (11), and hepatocellular carcinoma (12), and thus the findings on FBXO5 in this research need to be further validated in other tumor types. Third, only bioinformatic analyses were conducted to access the correlations between FBXO5 expression and prognostic and immunological features based upon public databases. Therefore, the experimental verification should be performed in the future to overcome this issue.

## Conclusion

In conclusion, the present research demonstrated that FBXO5 was a potential oncogene that could serve as an independent prognostic biomarker in various cancer types. FBXO5 expression was also correlated with DNA methylation modification, TME, infiltration of immune cells, immune-related genes, immune checkpoints, MSI, TMB, and MMR. These discoveries expanded the knowledge about the roles of FBXO5 in tumorigenesis and progression, proposing new insights for personalized cancer immunotherapy. Prospective studies focusing on tumor immunity and FBXO5 expression may be beneficial in providing a definite answer, thereby facilitating the development of an immunotherapy approach targeting FBXO5 for tumor in the future.

## Data Availability Statement

The datasets presented in this study can be found in online repositories. The names of the repository/repositories and accession number(s) can be found in the article/**Supplementary Material**.

## Ethics Statement

Ethical review and approval was not required for the study on human participants in accordance with the local legislation and institutional requirements. Written informed consent for participation was not required for this study in accordance with the national legislation and the institutional requirements.

## Author Contributions

PL collected and interpreted the data, organized the illustrations, and wrote the first version of the manuscript. XW collected and processed the data, and contributed to revising the manuscript. LP undertook statistical examination. BH reviewed the manuscript and made valuable academic suggestions. ZH provided the overall guidance for the study. All authors read and approved the final manuscript.

## Funding

This work was supported by the Major Program of National Key Research and Development Project (2020YFA0112600, 2019YFA0801502), National Natural Science Foundation of China (82103095, 82104242, 82173019), Jiangxi Provincial Natural Science Foundation (20212ACB206033), the Project of Shanghai Science and Technology Commission (19140902900), Program of Shanghai Academic/Technology Research Leader (20XD1434000), and Peak Disciplines (Type IV) of Institutions of Higher Learning in Shanghai.

## Conflict of Interest

The authors declare that the research was conducted in the absence of any commercial or financial relationships that could be construed as a potential conflict of interest.

## Publisher’s Note

All claims expressed in this article are solely those of the authors and do not necessarily represent those of their affiliated organizations, or those of the publisher, the editors and the reviewers. Any product that may be evaluated in this article, or claim that may be made by its manufacturer, is not guaranteed or endorsed by the publisher.

## Abbreviations

ACC, adrenocortical carcinoma; BLCA, bladder urothelial carcinoma; BRCA, breast invasive carcinoma; CESC, cervical squamous cell carcinoma and endocervical adenocarcinoma; CHOL, cholangiocarcinoma; COAD, colon adenocarcinoma; DLBC, lymphoid neoplasm diffuse large B-cell lymphoma; ESCA, esophageal carcinoma; GBM, glioblastoma; LGG, brain lower grade glioma; HNSC, head and neck squamous cell carcinoma; KICH, kidney chromophobe; KIRC, kidney renal clear cell carcinoma; KIRP, kidney renal papillary cell carcinoma; LAML, acute myeloid leukemia; LIHC, liver hepatocellular carcinoma; LUAD, lung adenocarcinoma; LUSC, lung squamous cell carcinoma; MESO, mesothelioma; OV, ovarian serous cystadenocarcinoma; PAAD, pancreatic adenocarcinoma; PCPG, pheochromocytoma and paraganglioma; PRAD, prostate adenocarcinoma; READ, rectum adenocarcinoma; SARC, sarcoma; SKCM, skin cutaneous melanoma; STAD, stomach adenocarcinoma; TGCT, testicular germ cell tumors; THCA, thyroid carcinoma; THYM, thymoma; UCEC, uterine corpus endometrial carcinoma; UCS, uterine carcinosarcoma; UVM, uveal melanoma.
