# Supplementary figures and images for: Prognostic Significance and Immunological Role of FBXO5 in Human Cancers: A Systematic Pan-Cancer Analysis
Source: Front Immunol. 2022 Jun 3;13:901784. doi: 10.3389/fimmu.2022.901784 (PMC9203914; doi:10.3389/fimmu.2022.901784)

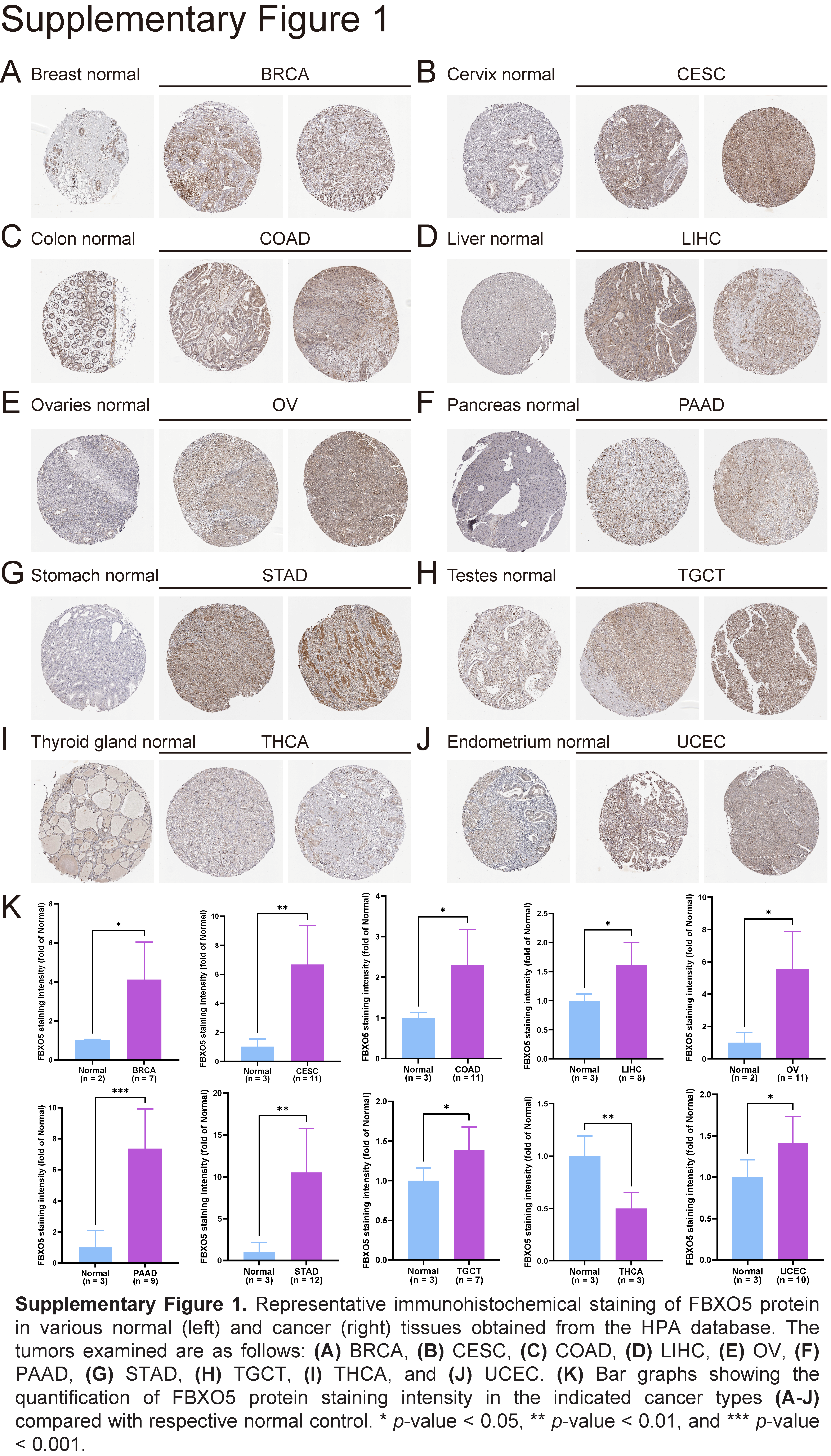

Supplement: Supplementary file 1 [file Image_1.tiff]
