# Supplementary material for: Prognostic Significance and Immunological Role of FBXO5 in Human Cancers: A Systematic Pan-Cancer Analysis
Source: Front Immunol. 2022 Jun 3;13:901784. doi: 10.3389/fimmu.2022.901784 (PMC9203914; doi:10.3389/fimmu.2022.901784)
Supplement: Supplementary file 9 [file DataSheet_1.pdf]

**Table S1.** Summary of Spearman's correlation between FBXO5 expression and drug resistance or sensitivity (IC50 value) in cancer cell lines based upon the GDSC2 dataset.

| No. | Drug name | Correlation | *p*-value | Label |
|---|---|---|---|---|
| 1 | ERK_2440 | 0.082539432 | 0.026152616 | positive |
| 2 | PD0325901 | 0.104192562 | 0.003154309 | positive |
| 3 | ERK_6604 | 0.124034271 | 0.000810424 | positive |
| 4 | SCH772984 | 0.177382097 | 4.43E-07 | positive |
| 5 | Selumetinib | 0.187303327 | 3.71E-07 | positive |
| 6 | Trametinib | 0.245383684 | 5.56E-12 | positive |
| 7 | BMS-754807 | 0.3329105 | 0.027236223 | positive |
| **No.** | **Drug name** | **Correlation** | ***p*-value** | **Label** |
| 1 | BI-2536 | -0.500302024 | 0.000634551 | negative |
| 2 | Tozasertib | -0.497720365 | 0.000318912 | negative |
| 3 | MK-1775 | -0.318515525 | 1.60E-19 | negative |
| 4 | Venetoclax | -0.309405445 | 3.61E-18 | negative |
| 5 | MK-8776 | -0.29746105 | 1.05E-16 | negative |
| 6 | Wee1 Inhibitor | -0.291228999 | 3.63E-16 | negative |
| 7 | Temozolomide | -0.288061682 | 9.69E-16 | negative |
| 8 | AZD7762 | -0.28779851 | 5.83E-16 | negative |
| 9 | Teniposide | -0.284267651 | 5.82E-15 | negative |
| 10 | Zoledronate | -0.28255679 | 8.57E-15 | negative |
| 11 | AMG-319 | -0.280042932 | 7.14E-15 | negative |
| 12 | Vorinostat | -0.277224577 | 8.68E-15 | negative |
| 13 | MIRA-1 | -0.276678853 | 1.31E-14 | negative |
| 14 | Alisertib | -0.274576337 | 4.85E-14 | negative |
| 15 | Daporinad | -0.272947218 | 6.44E-08 | negative |
| 16 | MIM1 | -0.269020486 | 6.96E-14 | negative |
| 17 | Olaparib | -0.268724708 | 5.27E-14 | negative |
| 18 | AZD5991 | -0.267230384 | 2.85E-13 | negative |
| 19 | Leflunomide | -0.266355604 | 2.95E-13 | negative |
| 20 | Navitoclax | -0.265128902 | 1.75E-13 | negative |
| 21 | ABT737 | -0.262400021 | 3.04E-13 | negative |
| 22 | VE821 | -0.262124263 | 4.15E-13 | negative |
| 23 | Cisplatin | -0.261407424 | 2.04E-13 | negative |
| 24 | YK-4-279 | -0.260689804 | 4.22E-13 | negative |
| 25 | WEHI-539 | -0.254895925 | 1.42E-12 | negative |
| 26 | EPZ5676 | -0.254638458 | 2.82E-13 | negative |
| 27 | Vincristine | -0.25337745 | 5.20E-12 | negative |
| 28 | IWP-2 | -0.253192281 | 4.56E-12 | negative |
| 29 | I-BRD9 | -0.25227519 | 2.45E-12 | negative |
| 30 | EPZ004777 | -0.251183677 | 6.01E-13 | negative |
| 31 | VE-822 | -0.250008189 | 8.29E-12 | negative |
| 32 | AZD6738 | -0.249760665 | 4.09E-12 | negative |
| 33 | Nelarabine | -0.249564419 | 9.34E-12 | negative |

| | | | | |
|---|---|---|---|---|
| 34 | Niraparib | -0.246431236 | 1.67E-11 | negative |
| 35 | Pyridostatin | -0.245377001 | 1.05E-11 | negative |
| 36 | Vinblastine | -0.244965295 | 1.14E-11 | negative |
| 37 | AZD1208 | -0.244711484 | 2.40E-11 | negative |
| 38 | GSK2578215A | -0.242265895 | 1.82E-11 | negative |
| 39 | PD173074 | -0.241242488 | 1.85E-11 | negative |
| 40 | PFI3 | -0.241126959 | 4.60E-11 | negative |
| 41 | BIBR-1532 | -0.240251056 | 2.96E-11 | negative |
| 42 | Vinorelbine | -0.239359757 | 3.52E-11 | negative |
| 43 | Sabutoclax | -0.238763993 | 7.63E-11 | negative |
| 44 | Sorafenib | -0.238535268 | 3.24E-11 | negative |
| 45 | Irinotecan | -0.238059093 | 9.59E-12 | negative |
| 46 | Mitoxantrone | -0.23771499 | 8.74E-11 | negative |
| 47 | BMS-345541 | -0.237254378 | 9.52E-11 | negative |
| 48 | PCI-34051 | -0.236608179 | 1.07E-10 | negative |
| 49 | KRAS (G12C) Inhibitor-12 | -0.235247575 | 1.38E-10 | negative |
| 50 | Cyclophosphamide | -0.235134551 | 7.44E-11 | negative |
| 51 | Ruxolitinib | -0.234712892 | 1.52E-10 | negative |
| 52 | CZC24832 | -0.234235032 | 1.66E-10 | negative |
| 53 | PRIMA-1MET | -0.233868531 | 1.78E-10 | negative |
| 54 | Podophyllotoxin bromide | -0.233532951 | 1.89E-10 | negative |
| 55 | Picolinici-acid | -0.23086797 | 3.07E-10 | negative |
| 56 | Gallibiscoquinazole | -0.230116058 | 3.52E-10 | negative |
| 57 | AZ960 | -0.225865061 | 8.14E-10 | negative |
| 58 | Elephantin | -0.225625001 | 7.83E-10 | negative |
| 59 | Carmustine | -0.225068335 | 8.64E-10 | negative |
| 60 | Wnt-C59 | -0.225009222 | 8.73E-10 | negative |
| 61 | RVX-208 | -0.222864237 | 1.30E-09 | negative |
| 62 | Nilotinib | -0.221872088 | 7.31E-10 | negative |
| 63 | Eg5_9814 | -0.221295272 | 1.85E-09 | negative |
| 64 | GSK1904529A | -0.220385256 | 3.11E-10 | negative |
| 65 | P22077 | -0.220156729 | 1.13E-09 | negative |
| 66 | ML323 | -0.21833603 | 2.77E-09 | negative |
| 67 | AGI-5198 | -0.217800019 | 1.71E-09 | negative |
| 68 | IAP_5620 | -0.215505112 | 4.81E-09 | negative |
| 69 | AGI-6780 | -0.215151074 | 4.75E-09 | negative |
| 70 | Camptothecin | -0.213664573 | 9.37E-10 | negative |
| 71 | ULK1_4989 | -0.21305363 | 7.06E-09 | negative |
| 72 | LY2109761 | -0.213053234 | 6.74E-09 | negative |
| 73 | PAK_5339 | -0.212716409 | 7.29E-09 | negative |
| 74 | AT13148 | -0.212010447 | 5.48E-09 | negative |
| 75 | JAK_8517 | -0.211772847 | 8.33E-09 | negative |
| 76 | Entinostat | -0.210943435 | 8.29E-09 | negative |
| 77 | IRAK4_4710 | -0.207610973 | 1.64E-08 | negative |

| | | | | |
|---|---|---|---|---|
| 78 | GSK343 | -0.20725207 | 1.78E-08 | negative |
| 79 | Talazoparib | -0.207128326 | 1.16E-08 | negative |
| 80 | LJI308 | -0.205700675 | 1.53E-08 | negative |
| 81 | LGK974 | -0.20555479 | 4.42E-09 | negative |
| 82 | Topotecan | -0.20521324 | 2.41E-08 | negative |
| 83 | BPD-00008900 | -0.20270901 | 2.19E-08 | negative |
| 84 | Cytarabine | -0.19918628 | 3.85E-08 | negative |
| 85 | GDC0810 | -0.197515461 | 5.03E-08 | negative |
| 86 | Savolitinib | -0.196568716 | 5.96E-08 | negative |
| 87 | Tamoxifen | -0.19579327 | 6.08E-08 | negative |
| 88 | JAK1_8709 | -0.195093144 | 1.17E-07 | negative |
| 89 | I-BET-762 | -0.195018807 | 1.20E-07 | negative |
| 90 | Epirubicin | -0.194775494 | 7.73E-08 | negative |
| 91 | Paclitaxel | -0.194057112 | 7.83E-08 | negative |
| 92 | Fludarabine | -0.193305232 | 1.53E-07 | negative |
| 93 | Oxaliplatin | -0.191596385 | 4.58E-14 | negative |
| 94 | Pevonedistat | -0.191410651 | 1.35E-07 | negative |
| 95 | Gemcitabine | -0.191139022 | 1.26E-07 | negative |
| 96 | GSK591 | -0.187803636 | 2.56E-07 | negative |
| 97 | CDK9_5038 | -0.185709769 | 4.76E-07 | negative |
| 98 | IGF1R_3801 | -0.185029592 | 5.25E-07 | negative |
| 99 | AZD4547 | -0.184597183 | 3.38E-07 | negative |
| 100 | UMI-77 | -0.18451651 | 3.67E-07 | negative |
| 101 | Crizotinib | -0.184427281 | 4.96E-07 | negative |
| 102 | Foretinib | -0.184312453 | 3.92E-07 | negative |
| 103 | Mirin | -0.18144279 | 8.60E-07 | negative |
| 104 | LCL161 | -0.180676618 | 9.74E-07 | negative |
| 105 | Sinularin | -0.177323252 | 1.53E-06 | negative |
| 106 | Fulvestrant | -0.174620056 | 1.20E-11 | negative |
| 107 | XAV939 | -0.17319995 | 2.68E-06 | negative |
| 108 | AZD5438 | -0.172768409 | 2.84E-06 | negative |
| 109 | NVP-ADW742 | -0.172292811 | 2.11E-06 | negative |
| 110 | OTX015 | -0.170362303 | 3.91E-06 | negative |
| 111 | Linsitinib | -0.169698218 | 1.38E-06 | negative |
| 112 | Telomerase Inhibitor IX | -0.168424923 | 3.57E-06 | negative |
| 113 | MG-132 | -0.168408727 | 2.65E-06 | negative |
| 114 | Docetaxel | -0.167899544 | 1.67E-10 | negative |
| 115 | AZD5582 | -0.162045083 | 1.35E-05 | negative |
| 116 | WIKI4 | -0.159627078 | 1.15E-05 | negative |
| 117 | Dinaciclib | -0.158693116 | 1.76E-05 | negative |
| 118 | Dabrafenib | -0.157986852 | 1.33E-05 | negative |
| 119 | CDK9_5576 | -0.157693917 | 2.00E-05 | negative |
| 120 | Staurosporine | -0.156181181 | 1.36E-05 | negative |
| 121 | Sepantronium bromide | -0.154834267 | 2.08E-05 | negative |

| | | | | |
|---|---|---|---|---|
| 122 | Buparlisib | -0.153893862 | 2.34E-05 | negative |
| 123 | MK-2206 | -0.151472493 | 2.53E-05 | negative |
| 124 | GSK2606414 | -0.151286509 | 4.64E-05 | negative |
| 125 | Bortezomib | -0.150530082 | 3.36E-05 | negative |
| 126 | TAF1_5496 | -0.149718309 | 5.18E-05 | negative |
| 127 | AZD5153 | -0.146246574 | 7.74E-05 | negative |
| 128 | PRT062607 | -0.145402504 | 8.43E-05 | negative |
| 129 | Obatoclax Mesylate | -0.142030926 | 0.000123122 | negative |
| 130 | BDP-00009066 | -0.141532097 | 0.000101682 | negative |
| 131 | MN-64 | -0.138727368 | 0.000177097 | negative |
| 132 | AZD5363 | -0.138578355 | 0.000142016 | negative |
| 133 | Palbociclib | -0.13791501 | 0.000128607 | negative |
| 134 | AZ6102 | -0.132145101 | 0.000304078 | negative |
| 135 | Cediranib | -0.126998936 | 0.000493978 | negative |
| 136 | Dactinomycin | -0.122387614 | 2.67E-06 | negative |
| 137 | Afuresertib | -0.121198559 | 0.000895547 | negative |
| 138 | Luminespib | -0.117954879 | 0.000823354 | negative |
| 139 | OF-1 | -0.117237334 | 0.001554226 | negative |
| 140 | Uprosertib | -0.114479387 | 1.05E-05 | negative |
| 141 | Ipatasertib | -0.110026545 | 0.002567069 | negative |
| 142 | Rapamycin | -0.103782023 | 0.004656729 | negative |
| 143 | Alpelisib | -0.103071605 | 0.003496358 | negative |
| 144 | AZD1332 | -0.102851814 | 0.005539496 | negative |
| 145 | Entospletinib | -0.099933008 | 0.007044311 | negative |
| 146 | Dihydrorotenone | -0.094396799 | 0.011045822 | negative |
| 147 | 5-Fluorouracil | -0.092201702 | 0.008985375 | negative |
| 148 | Nutlin-3a (-) | -0.09177498 | 0.010889233 | negative |
| 149 | Ibrutinib | -0.088225125 | 0.017733709 | negative |
| 150 | Afatinib | -0.086198805 | 0.014673962 | negative |
| 151 | AZD3759 | -0.083943365 | 0.020896558 | negative |
| 152 | Dactolisib | -0.080667642 | 0.027271299 | negative |
| 153 | Erlotinib | -0.074826671 | 0.040628966 | negative |

| **No.** | **Drug name** | **Correlation** | ***p*-value** | **Label** |
|---|---|---|---|---|
| 1 | ZM447439 | -0.19553654 | 0.187774855 | ns |
| 2 | RO-3306 | -0.149261832 | 0.311278209 | ns |
| 3 | NU7441 | -0.127312673 | 0.393777526 | ns |
| 4 | Axitinib | -0.118469388 | 0.417515923 | ns |
| 5 | VSP34_8731 | -0.067482249 | 0.069185491 | ns |
| 6 | PLX-4720 | -0.059709486 | 0.092696145 | ns |
| 7 | BMS-536924 | -0.058117276 | 0.112007875 | ns |
| 8 | WZ4003 | -0.057188327 | 0.123680003 | ns |
| 9 | KU-55933 | -0.052757273 | 0.72174586 | ns |
| 10 | JQ1 | -0.050763182 | 0.734713524 | ns |
| 11 | GSK269962A | -0.046026921 | 0.756072145 | ns |

| | | | | |
|---|---|---|---|---|
| 12 | AZD6482 | -0.045097132 | 0.763415789 | ns |
| 13 | Dasatinib | -0.044894826 | 0.217277184 | ns |
| 14 | Osimertinib | -0.039863347 | 0.275895698 | ns |
| 15 | Lapatinib | -0.03386753 | 0.353047651 | ns |
| 16 | Gefitinib | -0.033074574 | 0.366039973 | ns |
| 17 | OSI-027 | -0.022034015 | 0.722597479 | ns |
| 18 | SB505124 | -0.014624506 | 0.924038004 | ns |
| 19 | Taselisib | -0.014123399 | 0.689808231 | ns |
| 20 | AZD8186 | -0.010462521 | 0.774980423 | ns |
| 21 | Pictilisib | -0.008487774 | 0.814928314 | ns |
| 22 | GNE-317 | -0.003195077 | 0.93108951 | ns |
| 23 | Ulixertinib | 0.02288514 | 0.376569435 | ns |
| 24 | AZD2014 | 0.024624109 | 0.507690109 | ns |
| 25 | Sapitinib | 0.031067416 | 0.380184141 | ns |
| 26 | Acetalax | 0.049966778 | 0.178675472 | ns |
| 27 | VX-11e | 0.06173859 | 0.092638064 | ns |
| 28 | AZD8055 | 0.063546298 | 0.545084518 | ns |
| 29 | Doramapimod | 0.092568609 | 0.540624669 | ns |
| 30 | Ribociclib | 0.121060746 | 0.422881883 | ns |
| 31 | SB216763 | 0.1454597 | 0.164164175 | ns |
| 32 | PF-4708671 | 0.145570994 | 0.323528874 | ns |

“ns” denotes no significance.
