# Supplementary material for: Prognostic Significance and Immunological Role of FBXO5 in Human Cancers: A Systematic Pan-Cancer Analysis
Source: Front Immunol. 2022 Jun 3;13:901784. doi: 10.3389/fimmu.2022.901784 (PMC9203914; doi:10.3389/fimmu.2022.901784)
Supplement: Supplementary file 10 [file DataSheet_2.pdf]

Table S2. Summary of the association of FBXO5 expression with ImmuneScore and prognosis in pan-cancer.

Cancer type	FBXO5 expression	Immune Score	Prognostic value			
			OS survival	DSS survival	DFI survival	PFI survival
ACC	↑	▲	▲	▲	ns	▲
BLCA	↑	ns	ns	ns	ns	ns
BRCA	↑	ns	ns	ns	ns	ns
CESC	↑	▲	ns	ns	ns	ns
CHOL	↑	ns	ns	ns	ns	ns
COAD	↑	ns	ns	ns	ns	ns
DLBC	↑	ns	ns	ns	ns	ns
ESCA	↑	▲	ns	▲	ns	ns
GBM	↑	▲	ns	ns	×	●
HNSC	↑	ns	ns	ns	ns	ns
KICH	↓	ns	▲	▲	ns	▲
KIRC	↑	●	ns	ns	ns	ns
KIRP	ns	▲	▲	▲	▲	▲
LAML	↓	▲	ns	×	×	×
LGG	↑	ns	▲	▲	ns	ns
LIHC	↑	ns	▲	▲	▲	▲
LUAD	↑	ns	▲	▲	ns	ns
LUSC	↑	▲	ns	ns	ns	ns
MESO	×	ns	▲	▲	ns	▲
OV	↑	ns	ns	ns	ns	ns
PAAD	↑	●	ns	ns	ns	ns
PCPG	ns	▲	ns	ns	ns	ns
PRAD	ns	ns	ns	ns	ns	ns
READ	↑	ns	●	ns	ns	ns
SARC	ns	▲	ns	▲	ns	ns
SKCM	↑	ns	ns	ns	×	ns
STAD	↑	▲	ns	ns	ns	ns
TGCT	↑	▲	ns	ns	ns	ns
THCA	↓	▲	ns	ns	●	ns
THYM	↑	ns	ns	ns	×	ns
UCEC	↑	▲	ns	ns	ns	ns
UCS	↑	▲	ns	ns	ns	ns
UVM	×	ns	ns	ns	×	ns

↑ denotes increased expression of FBXO5 in tumor tissues compared with normal tissues; ↓ denotes decreased expression of FBXO5 in tumor tissues compared with normal tissues; ▲ denotes the negative correlation between FBXO5 expression and ImmuneScore or survival time in cancer patients; ● denotes the positive correlation between FBXO5 expression and ImmuneScore or survival time in cancer patients; "ns" denotes no significance; × denotes that statistical analysis is not available; OS, overall survival; DSS, disease-specific survival; DFI, disease-free interval; PFI, progression-free interval; ACC, adrenocortical carcinoma; BLCA, bladder urothelial carcinoma; BRCA, breast invasive carcinoma; CESC, cervical squamous cell carcinoma and endocervical adenocarcinoma; CHOL, cholangiocarcinoma; COAD, colon adenocarcinoma; DLBC, lymphoid neoplasm diffuse large B-cell lymphoma; ESCA, esophageal carcinoma; GBM, glioblastoma; LGG, brain lower grade glioma; HNSC, head and neck squamous cell carcinoma; KICH, kidney chromophobe; KIRC, kidney renal clear cell carcinoma; KIRP, kidney renal papillary cell carcinoma; LAML, acute myeloid leukemia; LIHC, liver hepatocellular carcinoma; LUAD, lung adenocarcinoma; LUSC, lung squamous cell carcinoma; MESO, mesothelioma; OV, ovarian serous cystadenocarcinoma; PAAD, pancreatic adenocarcinoma; PCPG, pheochromocytoma and paraganglioma; PRAD, prostate adenocarcinoma; READ, rectum adenocarcinoma; SARC, sarcoma; SKCM, skin cutaneous melanoma; STAD, stomach adenocarcinoma; TGCT, testicular germ cell tumors; THCA, thyroid carcinoma; THYM, thymoma; UCEC, uterine corpus endometrial carcinoma; UCS, uterine carcinosarcoma; UVM, uveal melanoma.
